# Function of ORFC of the polyketide synthase gene cluster on fatty acid accumulation in *Schizochytrium limacinum* SR21

**DOI:** 10.1186/s13068-021-02014-9

**Published:** 2021-07-23

**Authors:** Yanyan Shi, Zhen Chen, Yixin Li, Xingyu Cao, Lijie Yang, Yiyuan Xu, Zhipeng Li, Ning He

**Affiliations:** 1 Department of Chemical and Biochemical Engineering, College of Chemistry and Chemical Engineering, Xiamen University, Xiamen, 361005 China; 2 College of Life Science, Xinyang Normal University, Xinyang, 464000 China; 3 College of Food and Biological Engineering, Jimei University, Xiamen, 361021 China

**Keywords:** Polyunsaturated fatty acids, Dehydratase, Enoyl reductase, Transcriptomics, Metabolomics, *Schizochytrium limacinum*

## Abstract

**Background:**

As a potential source of polyunsaturated fatty acids (PUFA), *Schizochytrium* sp. has been widely used in industry for PUFA production. Polyketide synthase (PKS) cluster is supposed to be the primary way of PUFA synthesis in *Schizochytrium* sp. As one of three open reading frames (ORF) in the PKS cluster, ORFC plays an essential role in fatty acid biosynthesis. However, the function of domains in ORFC in the fatty acid synthesis of *Schizochytrium* sp. remained unclear.

**Results:**

In this study, heterologous expression and overexpression were carried out to study the role of ORFC and its domains in fatty acid accumulation. Firstly, ORFC was heterologously expressed in yeast which increased the PUFA content significantly. Then, the dehydratase (DH) and enoyl reductase (ER) domains located on ORFC were overexpressed in *Schizochytrium limacinum* SR21, respectively. Fatty acids profile analysis showed that the contents of PUFA and saturated fatty acid were increased in the DH and ER overexpression strains, respectively. This indicated that the DH and ER domains played distinct roles in lipid accumulation. Metabolic and transcriptomic analysis revealed that the pentose phosphate pathway and triacylglycerol biosynthesis were enhanced, while the tricarboxylic acid cycle and fatty acids oxidation were weakened in DH-overexpression strain. However, the opposite effect was found in the ER-overexpression strain.

**Conclusion:**

Therefore, ORFC was required for the biosynthesis of fatty acid. The DH domain played a crucial role in PUFA synthesis, whereas the ER domain might be related to saturated fatty acids (SFA) synthesis in *Schizochytrium limacinum* SR21. This research explored the role of ORFC in the PKS gene cluster in *Schizochytrium limacinum* and provided potential genetic modification strategies for improving lipid production and regulating PUFA and SFA content.

**Supplementary Information:**

The online version contains supplementary material available at 10.1186/s13068-021-02014-9.

## Background

Polyunsaturated fatty acids (PUFA), especially docosahexaenoic acid (DHA, 22:6Δ^4,7,10,13,16,19^) and eicosapentaenoic acid (EPA, 20:5Δ^5,8,11,14,17^) are essential for human health [1–4]. Industrial production on a large scale is currently being launched via biotechnological methods based on marine algae [5]. It is known that the polyketide synthase (PKS) pathway is the primary way to synthesize PUFA in *Schizochytrium* sp. [6]. Fatty acid synthases, which are large enzyme complexes with multiple catalytic domains, synthesize PUFA from acetyl units via the PKS pathway, as demonstrated by microalgal DHA production [6].

**Fig. 1:**
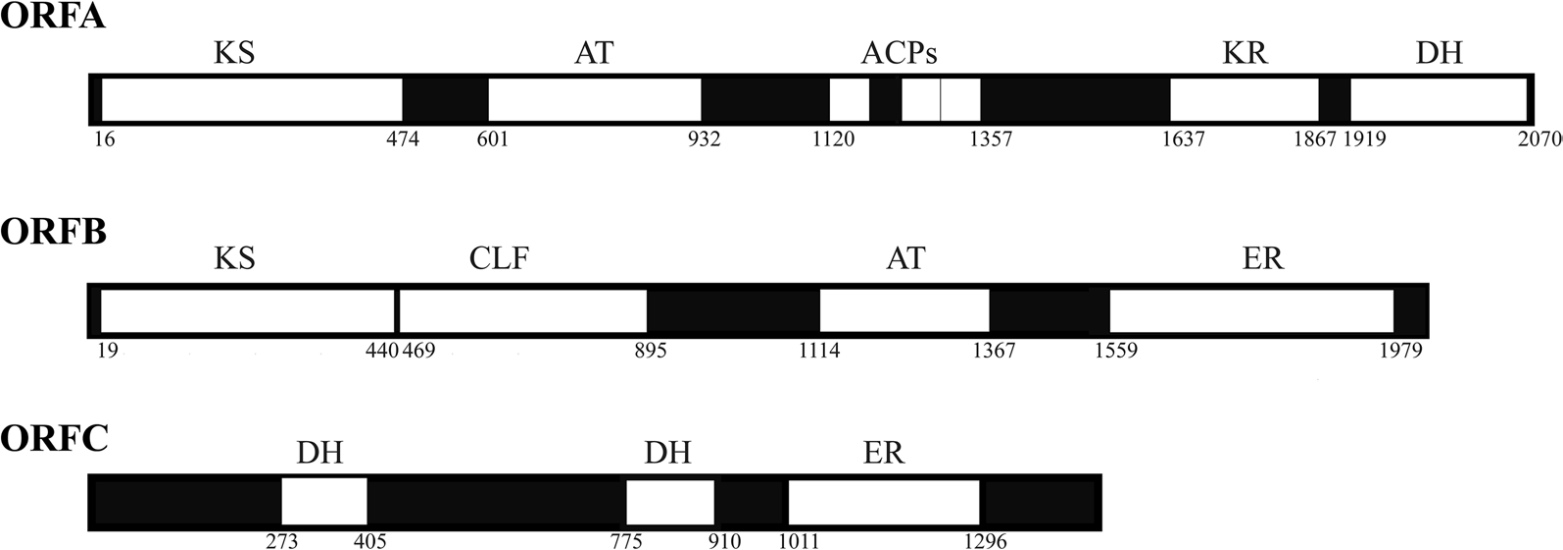
The domain organization of the PKS gene cluster. The numbers indicate the positions of the amino acids. *ACP* acyl carrier protein, *KS* 3-ketoacyl-ACP synthase, *CLF* chain length factor, *KR* 3-hydroxyacyl-ACP reductase, *DH* dehydratase; *ER* enoyl-ACP reductase, *MAT* malonyl acyl transferase. In this study, the first DH and ER domains of ORFC was investigated

Three genes encoding PKS were found in *Schizochytrium* sp. by Metz in 2001 [6]. The PKS pathway is composed of three open reading frames (ORFA, ORFB, and ORFC) that possess domain structures similar to fatty synthase (FAS), including dehydratase (DH), acyltransferase (AT), malonyl-CoA transacylase (MAT), β-ketoacyl synthase (KS), acyl carrier protein (ACP), ketoacyl reductase (KR), and enoyl reductase (ER) domains [7]. As illustrated in Fig. 1, the putative domains and active sites of the PKS gene cluster were identified by several online bioinformatic analysis tools, including BLAST (NCBI), SMART [8] and PKS/NRPS analysis [9]. Recently, research on the synthetic ability of PKS gene cluster to improve microbial strains and characterize their synthetic pathways has become a research hotspot [10–12]. Hayashi et al. investigated the function of ACPs and found that the number of activated ACPs increased the productivity of PUFA [13]. They also investigated the role of the KS domains in PUFA synthases by in vivo and in vitro analysis, and found that that two KS domains in subunit A and subunit C (KS_A_ and KS_C_) were verified to catalyze the condensation from C18-C20 and C20-C22, respectively [14].

DH domain, functional as FabA in *E. coli*, catalyze the dehydration of β-hydroxyacyl-ACP [15]. According to Xie et al., the DH domain of ORFC could catalyze the transformation of 3-hydroxyacyl-ACP to trans-2-decenoyl-ACP and then isomerize this product to generate 3-cis-decenoyl-ACP, which is a critical step in the PUFA biosynthesis [16]. To study the detailed mechanism for the production of PUFA, Hayashi et al. discovered that depending on the carbon chain length, the DH domains in subunit A and subunit C introduced either saturation or cis double bonds to growing acyl chains [17]. Similarly, in our previous work, the content of PUFA was decreased in *Schizochytrium* sp. by deleting the DH domain, indicating the DH domain was required for PUFA synthesis [18]. The ER domain is similar to FabI in *E. coli* and is capable of catalyzing double bond reduction during EPA biosynthesis in *Shewanella* via the PKS pathway [6]. Ling et al. found that the ORFB-ER played a key role in PUFA synthesis, and the ORFC-ER might be related to saturated fatty acids (SFA) synthesis by knocking out the ER domains on ORFB and ORFC, respectively [19]. However, the function of DH and ER domains still remains unknown.

Recently, metabolomic and transcriptomic analysis have been widely used in microalgae research. Lim et al. suggested that the increase in lipid accumulation was due to the decrease in lipid catabolism in the early stable phase in *Tetraselmis* sp. M8 with nitrogen source deficiency by transcriptomic and metabolomic analysis [20]. By performing ultraviolet mutagenesis on *Aurantiochytrium* sp., Liu et al. obtained strains with high yields of lipids and DHA, and further comparative transcriptomics analysis revealed that coenzyme A transferase, AT, ER, DH and methyltransferase might be the key genes responsible for increased DHA production in *Aurantiochytrium* [21]. These studies laid a foundation for us to explore the synthesis mechanism of PUFA in *Schizochytrium* sp. by using the omics method.

ORFC, which consists of two DH and one ER domains, is considered one of the critical regulators for enhancing fatty acid biosynthesis [22]. However, the function of ORFC in fatty acid synthesis of *Schizochytrium* sp. remains unclear. In this study, to investigate the role of ORFC in fatty acid accumulation, we constructed an ORFC heterologous expression strain in yeast and overexpressed these domains (located on ORFC) in *Schizochytrium limacinum* SR21. Gas chromatography/mass spectrometry (GC–MS) based metabolomic analysis and an RNA-seq analysis were conducted to explain the change in fatty acid composition in different strains. This study could provide more explanations and demonstrations for the role of ORFC in fatty acid synthesis.

## Results

### Effects of ORFC heterologous expression on fatty acid analysis in *S. cerevisiae*

To study the effects of ORFC on fatty acid accumulation, heterologous expression of ORFC was conducted in *S. cerevisiae* YSG50. As shown in Additional file 1: Fig. S2, single clones grew in the Ura-depletion plate, and ORFC gene was detected in the engineered strain while not in the wild-type strain by genomic PCR analysis, revealed that ORFC was successfully heterologously expressed in *S. cerevisiae* YSG50. As shown in Table 1, the total lipids (TL) yield had a slightly increase in the engineered strain compared to the wild-type strain. However, both the SFA and PUFA contents significantly increased by 33.24% and 125% in the engineered strain compared with the wild-type strain. C18:1 showed a significant decrease compared with the wild-type strain. Moreover, dihomo-gamma linolenic acid (DGLA), docosapentaenoic acid (DPA) and DHA were found in the engineered strain while none was produced in the wild-type strain. A higher ratio of PUFAs/SFAs was found in the engineered strain. These results indicated that the introduction of ORFC accelerated the conversion of C18 to C20, which in turn enables the synthesis of PUFA with more than 20 carbons in the yeast, such as DPA and DHA.

**Table 1:** Lipid profile of YSG50 (wild-type strain) and YSG50-C (engineered strain) after 3 days of fermentation

Lipids profile	YSG50	YSG50-C
Biomass (mg/L)	8166.67 ± 208.17	7200.00 ± 458.26*
TL (mg/L)	1190.67 ± 146.73	1265.33 ± 212.04
C14:0 (mg/L)	9.51 ± 0.69	30.57 ± 1.73*
C16:0 (mg/L)	282.85 ± 20.49	420.85 ± 45.77*
C18:0 (mg/L)	126.57 ± 21.09	106.77 ± 27.10
C18:1 (mg/L)	141.06 ± 28.21	73.56 ± 15.90*
DGLA (mg/L)	–	75.13 ± 14.28*
DPA (mg/L)	–	29.65 ± 3.92*
DHA (mg/L)	–	138.42 ± 16.51*
SFA (mg/L)	418.94 ± 40.34	558.19 ± 74.18*
PUFA (mg/L)	141.06 ± 28.21	316.75 ± 49.41*
PUFA/SFA	0.33 ± 0.03	0.57 ± 0.09*

*indicates *p* < 0.05. *TL* total lipids, *DGLA* dihomo-gamma linolenic acid, *DPA* docosapentaenoic acid, *DHA *docosahexaenoic acid, *SFA* saturated fatty acids, *PUFA* polyunsaturated fatty acids

### Construction of the DH/ER overexpressing strain

As shown in Additional file 1: Fig. S3C, a zeocin resistance gene was successfully detected but this gene was not detected in the wild-type strain. Moreover, qPCR analysis revealed that the transcription levels of DH and ER genes were significantly increased compared to the wild-type strain (Additional file 1: Fig. S4). These results indicated that DH and ER domains in *Schizochytrium limacinum* SR21 were successfully overexpressed, respectively.

### Effects of DH/ER domain overexpression on biomass and lipids accumulation

**Fig. 2:**
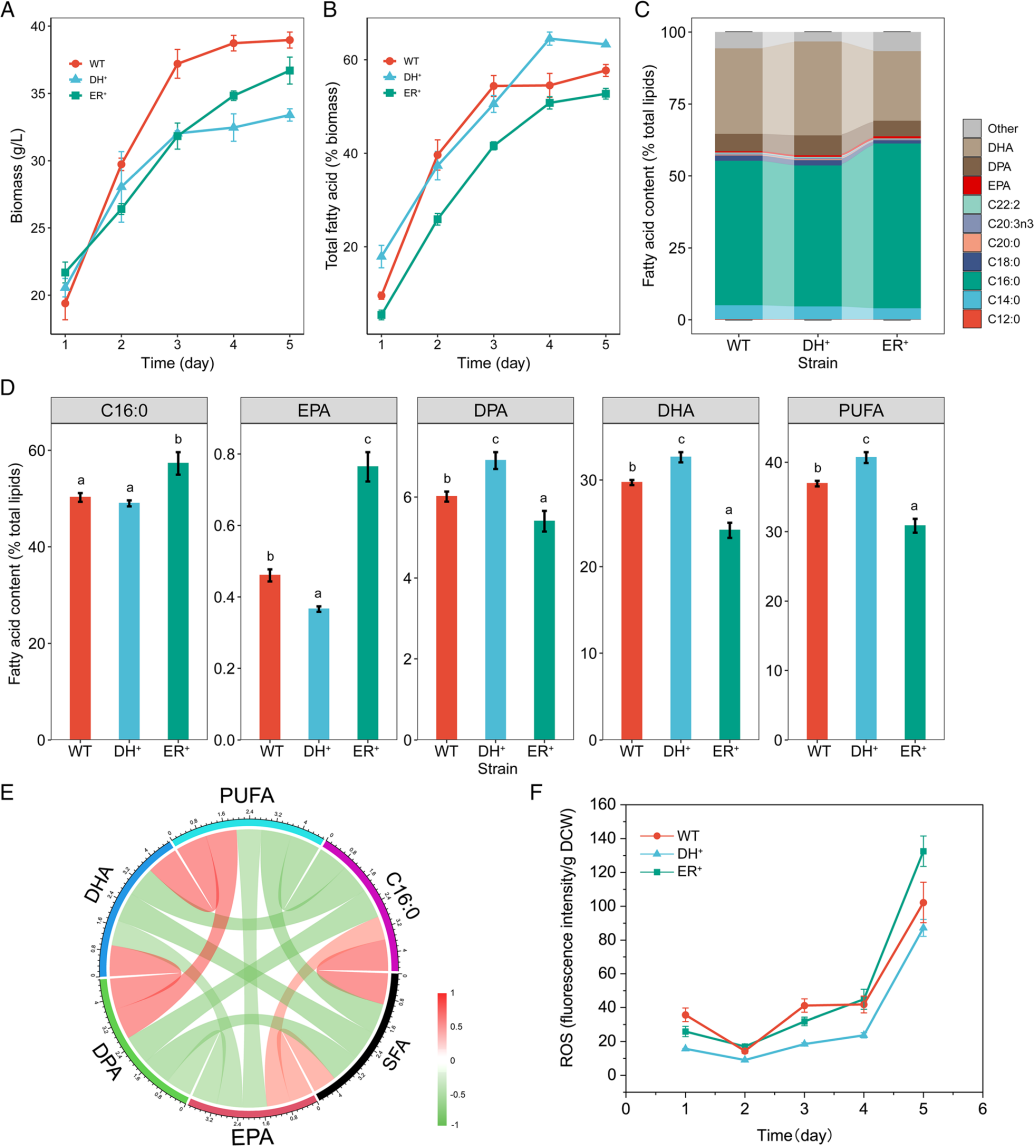
Biomass, lipid accumulation, and ROS levels of *Schizochytrium limacinum* SR21. **A** Biomass. **B** Percentage of the total fatty acid content to biomass. **C** Fatty acid composition of the total fatty acid at 5rd of fermentation.** D** Percentage of the primary fatty acids to total lipids after 5 days of fermentation. The different lowercase letters in each bar indicate a significant difference (*p* < 0.05) between different strains. **E **Spearman pairwise correlation globe among the different fatty acids. Each line represents a Spearman's correlation coefficient between two different fatty acids. Positive correlations are indicated in red, and the negative correlations are in green.** F** Reactive oxygen species (ROS) levels. Standard bars represent the standard deviation of triplicate samples. WT, ER^+^, and DH^+^ represent the wild-type strain, DH-overexpression strain, and ER-overexpression strain, respectively

As shown in Fig. 2A, the cell growth of both engineered strains was slower than that of the wild-type strain. The biomass of the DH-overexpression strain and ER-overexpression strain were 14.3% and 5.82% lower than that of the wild-type strain, respectively (*p* < 0.05). In addition, the TL content of the ER-overexpression strain was the lowest during the whole fermentation period among the strains (Fig. 2B). However, compared with the wild-type strain, the TL content of the DH-overexpression strain showed an increase of 9.7% (*p* < 0.05). These results suggested that overexpression of the ER domain had suppressive effects on biomass and lipid production. Both of engineered strains resulted in similar biomass inhibition, but the DH-overexpression strain could cause an increase in lipid production.

### Effects of DH/ER domain overexpression on fatty acid composition

Cells were collected to analyze fatty acid compositions on the final day of fermentation (the 5th day) when the lipid accumulation reached its highest level (Fig. 2B). As shown in Fig. 2C, D, overexpression of the ER and DH domains had a different and significant influence on fatty acid compositions. Compared to the wild-type strain, the DH-overexpression strain showed a 20.5% decrease in EPA content (*p* < 0.05), while the levels of DPA、DHA and PUFA increased by 14.8%, 9.8%, and 10.2% (*p* < 0.05), respectively. Conversely, in the ER-overexpression strain, EPA content increased by 66% (*p* < 0.05), while the levels of DPA, DHA and PUFA decreased by 10.1%, 18.6%, and 16.5% (*p* < 0.05), respectively.

Then, the correlations among the fatty acids were evaluated by using Spearman pairwise analysis. As shown in Fig. 2E, six fatty acids were divided into two categories. Interestingly, we found that there were significant positive correlations among C16:0, EPA and SFA. DHA, DPA and PUFA were also found to be positively correlated. However, any two fatty acids of the two categories were negatively correlated. As a result, we speculated that EPA synthesis might be related to SFA accumulation. However, additional research is required to confirm these findings.

### Effects of cellular reactive oxygen species (ROS) in DH/ER overexpression strains

The contents of intracellular ROS in *Schizochytrium limacinum* SR21 at 1-day intervals were determined during the entire fermentation process. As shown in Fig. 2F, cells of the three strains maintained low ROS levels during the initial stage (< 3 days), which might be related to the nutrient-rich medium [23]. From 3 days to the end of fermentation, the ROS levels of all strains increased significantly with the depletion of nutrients and the rapid accumulation of fatty acids. Additionally, the ROS level of the DH-overexpression strain remained low in comparison to the other strains throughout the fermentation period, reaching 118.7 at the end point of fermentation (5th day). Nonetheless, ROS levels remained higher in the ER-overexpression strain than in the other strains for the entire 5-day fermentation period.

### Metabolite profile analysis of DH/ER overexpression strains

**Fig. 3:**
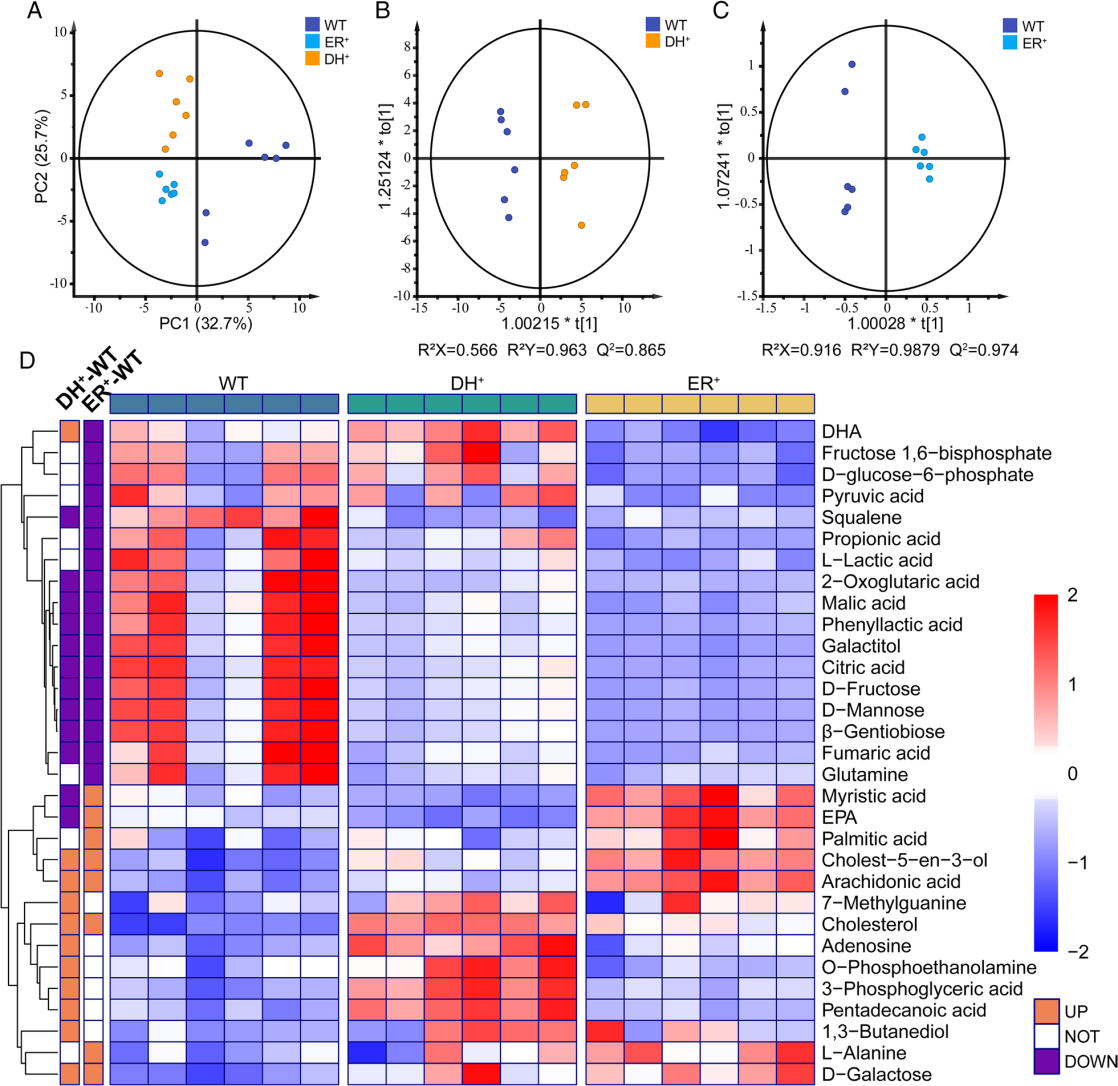
Metabolite analysis of the wild-type and engineered strains on the 3rd day of the fermentation process. **A** PCA score plot among the DH^+^, ER^+^ and WT strains. OPLS-DA score plots obtained from **B** WT vs. DH^+^ strains and **C** WT vs. ER^+^ strains. **D** Heatmap of normalized concentrations of 33 differential metabolites based on OPLS-DA results. Each column represents an individual. The normalized abundance values are depicted from blue to red, where red and blue indicate an increase and decrease in the metabolites, respectively. Yellow or purple entries indicate metabolites that are less or more abundant between different pairwise groups, respectively. WT, ER^+^, and DH^+^ represent the wild-type strain, the DH-overexpression strain, and the ER-overexpression strain, respectively

To uncover the intracellular fatty acid metabolism of all the engineered strains, GC–MS was adopted along with multivariate analysis. More than 51 putative intracellular metabolites were detected, identified and quantified in all samples on the 3rd day of fermentation, including fatty acids, organic acids, phosphorylated compounds, amino acids, and sugars. A principal component analysis (PCA) score plot was constructed to reveal the obvious separation of metabolites among the wild-type and engineered strains (Fig. 3A). Orthogonal partial least-squares discriminant analysis (OPLS-DA) pairwise comparison was performed to illustrate the remarkable diversity of the metabolic profiles between the wild-type strain and the engineered strains (Fig. 3B, C). Two OPLS-DA models had high Q^2^ values and low *p* values (less than 0.01) from CV-ANOVA. The permutation tests also suggested that the two OPLS-DA models had high predictive capability (Additional file 1: Fig. S5).

Differential metabolites were determined with variable importance in the projection (VIP) values greater than 1, and the *p*-values of less than 0.05 as statistically significant [24]. Based on these results, 26 and 25 differential metabolites were characterized between the wild-type strain and DH-overexpression strain and ER-overexpression strain, respectively. Differential metabolites in any two groups were all visually displayed in a heatmap plot (Fig. 3D).

To obtain a biochemical overview of differential metabolites, two metabolomics networks (Additional file 1: Fig. S6) were constructed and visually displayed using MetaMapp based on chemical similarity and enzymatic transformation. The resulting metabolic network mainly consisted of 8 distinctive clusters (TCA cycle, fatty acid metabolism, amino sugar and nucleotide sugar metabolism, glycolysis/pentose phosphate pathway (PPP pathway), galactose metabolism, amino acid metabolism, steroid biosynthesis, and nucleic acid metabolism). Moreover, the changing trends of some metabolites (DHA, EPA, palmitic acid (C16:0), pentadecanoic acid, myristic acid (C14:0), and 1-monopalmitin) were found to be different between the two engineered strains.

### Comparative transcriptomic analysis of engineered strains

**Fig. 4:**
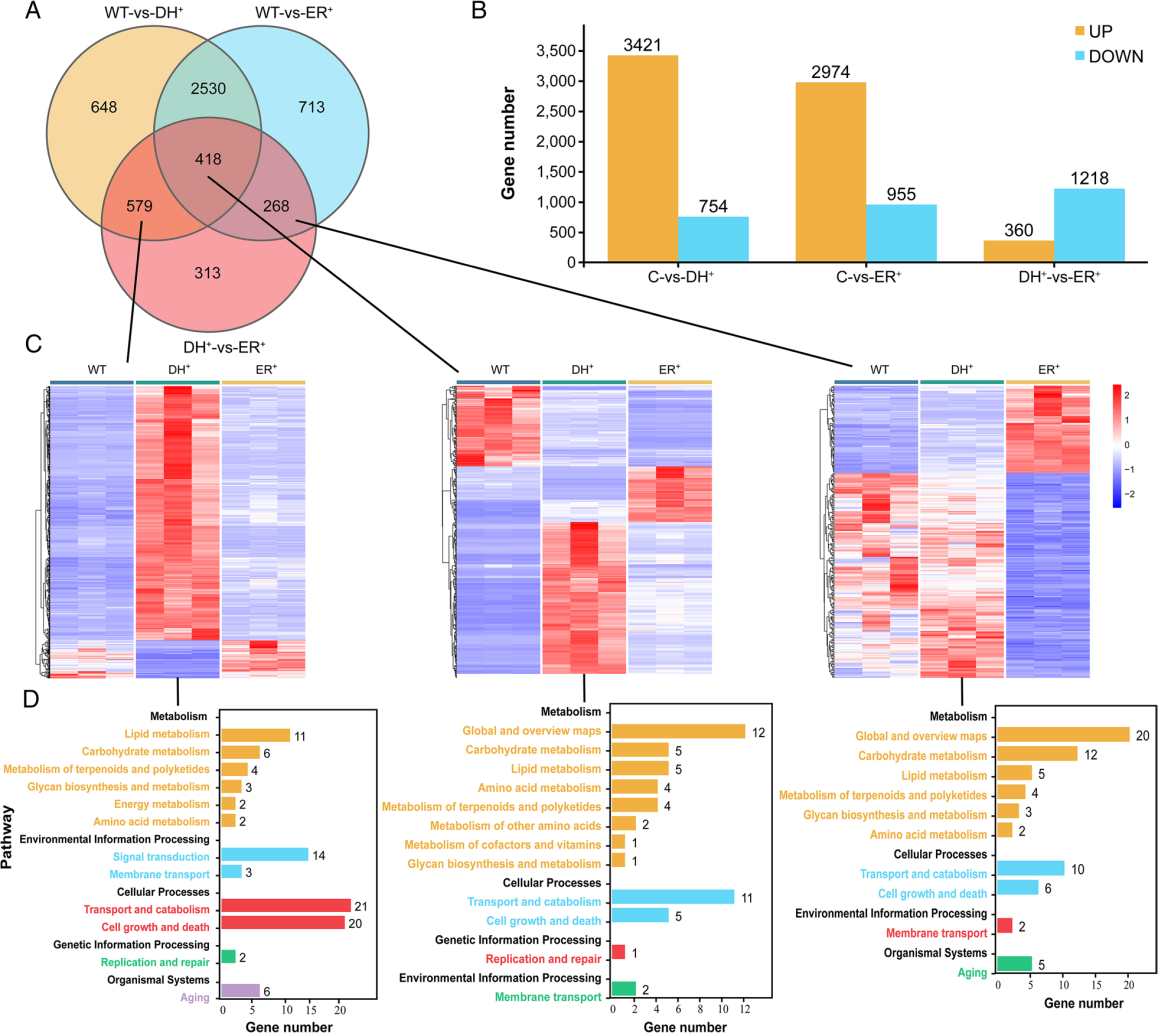
Transcriptomic analysis in the wild-type strain and engineered strains. **A** Venn diagram of the differentially expressed genes among different strains. **B** The number of up/down-regulated genes. **C** Heatmap of differentially expressed genes. **D** KEGG pathway enrichment analysis of differentially expressed genes among the different groups. WT, ER^+^, and DH^+^ represent the wild-type strain, the DH-overexpression strain, and the ER-overexpression strain, respectively

For a comprehensive understanding of the molecular mechanism underlying fatty acid changes in different domain overexpressing strains, RNA-seq analysis was conducted among the wild-type and engineered strains. In these strains, a total of 15,034 genes were identified corresponding to the reference genome of *Aurantiochytrium limacinum* ATCC MYA1381 [25]. A total of 5469 differentially expressed genes (DEGs) were identified among the wild-type and engineered strains under two criteria (|log_2_ (fold change)| > 1 and false discovery rate (FDR) < 0.01). There were 4175, 3929 and 1578 DEGs among the WT-DH^+^, WT-ER^+^ and DH^+^-ER^+^ groups, respectively, of which 3421, 2974 and 360 genes were upregulated and 754, 955 and 1218 genes were downregulated, respectively (Fig. 4A, B and Additional file 6: Table S7). There were 418 DEGs in commons in all 3 groups (Additional file 2: Table S3). Among these, 55 genes were downregulated in the DH-overexpression strains and upregulated in the ER-overexpression strains. In addition, 579 DEGs were observed only in the WT-DH^+^ and DH^+^-ER^+^ groups (Additional file 3: Table S4), and 268 DEGs were found only in the WT-ER^+^ and DH^+^-ER^+^ groups (Additional file 4: Table S5). The heatmap was illustrated to highlight these specific DEGs. As illustrated in Fig. 4C, the up- and down-regulation of these DEGs varied significantly among groups.

The Kyoto encyclopedia of genes and genomes (KEGG) pathway analysis was used to determine the significant enrichment pathways for DEGs (|log_2_ (fold change)| > 1 and *p* < 0.05). Figure 4D shows that 94 DEGs were significantly enriched in 12 pathways in the C-DH^+^ and DH^+^-ER^+^ groups, 28 of which were related to metabolism; 69 DEGs were enriched in 10 pathways in the C-ER^+^ and DH^+^-ER^+^ groups, 46 of which were related to metabolism; and 53 DEGs were enriched in 12 pathways in the three groups, 34 of which were related to metabolism. The pathways related to lipid metabolism included TAG biosynthesis and lipid oxidation. The pathways related to carbohydrate metabolism included the TCA cycle, glycolysis and inositol phosphate metabolism. The differentially expressed genes are shown as a heatmap in Additional file 1: Fig. S7, and the specific data are shown in Additional file 5: Table S6.

**Fig. 5:**
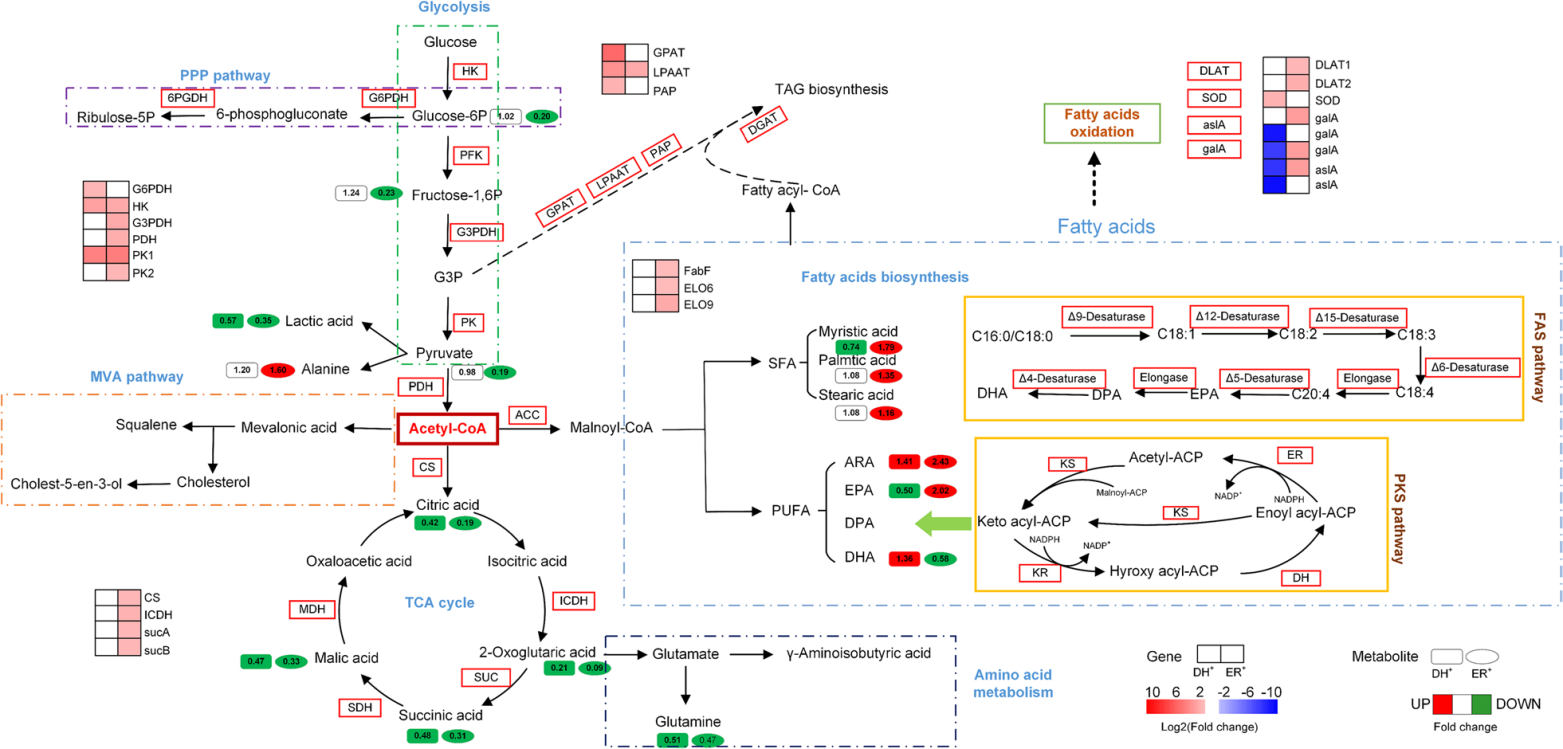
The specific metabolites and genes in the main metabolic pathways in the DH and ER overexpression strains. ER^+^, and DH^+^ represent the DH-overexpression strain and ER-overexpression strain, respectively. The red rectangle indicates key enzymes. Rectangle: DH-overexpression strain; oval: ER-overexpression strain. Red: increase (*p* < 0.05), green or blue: decrease (*p* < 0.05), white: no significant change (*p* > 0.05)

The differential metabolites and several key DEGs were further mapped to the major metabolic pathways (Fig. 5), which include the PPP, glycolysis, the mevalonate (MVA) pathway, the TCA cycle, TAG biosynthesis, amino acid metabolism, fatty acid biosynthesis and oxidation.

## Discussion

*Schizochytrium limacinum* is rich in PUFA, an alternative commercial lipid source. Bi et al. suggested that ORFC, one of the ORFs in the PKS cluster, played an essential role in PUFA (especially DHA) production [22]. In our study, heterologous expression of ORFC significantly increased TL and PUFA contents in *S. cerevisiae*, which suggested that ORFC played a vital role in PUFA biosynthesis. To investigate the function of ORFC for fatty acid biosynthesis, each domain of ORFC was overexpressed in *Schizochytrium limacinum* SR21. However, since a strain overexpressing the second DH domain exhibited no significant changes in fatty acid composition in comparison with the wild-type strain (*p* < 0.05, Additional file 1: Table S2), the first DH domain (named DH domain in this study) was deep investigated. Lipid profile analysis suggested that the PUFA (mainly DHA) content increased in the DH-overexpressed strain, whereas the SFA (mainly C16:0) content increased in the ER-overexpression strain. Our previous study indicated that deleting the DH domain and ER domain of ORFC could result in a decrease in the PUFA and SFA contents, respectively [19]. The DH domain of ORFC was also essential for the biosynthesis of PUFA through heterologous expression and site-mutagenesis in *E. coli* and *S. cerevisiae* [16, 26]. These results suggested that the DH and ER domains might exert distinct regulatory effects on the accumulation of PUFA and SFA. The DH-overexpression strain had a high PUFA content, whereas the ER-overexpression strain had a decreased PUFA content and an increased SFA content.

### Effects of acetyl-CoA, NADPH and TAG accumulation changes on fatty acids biosynthesis in engineered strains

Acetyl-CoA is an essential precursor for many pathways, such as the TCA cycle, amino acid biosynthesis, steroid biosynthesis, and fatty acid biosynthesis [27]. In the TCA cycle, the contents of 2-oxoglutaric acid, malic acid, succinic acid and citric acid were decreased, whereas ARA and DHA concentrations were increased in the DH-overexpression strain. This result suggested that the carbon flux could be redirected away from the TCA cycle and toward PUFA synthesis. Similar results were reported in which an enhanced acetyl-CoA supply could improve lipid production [28, 29]. However, the expression level of citrate synthase (*CS*), which catalyzes the conversion acetyl-CoA to citric acid in the TCA cycle, was upregulated significantly in the ER-overexpression strain. The same results were observed for a number of TCA cycle-related genes, including isocitrate dehydrogenase (*ICDH*), 2-oxoglutarate dehydrogenase E1 component (*sucA*), and the 2-oxoglutarate dehydrogenase E2 component (*sucB*). These findings might imply that acetyl-CoA was used to synthesize citrate acid rather than fatty acid biosynthesis, leading to a lower TL content in the ER-overexpression strain. Similarly, Deng et al. found that overexpression of the *CS* gene decreased the fatty acid level in *Chlamydomonas* [30].

Along with acetyl-CoA, the biosynthesis of fatty acids also requires a high concentration of NADPH [31, 32]. PPP was previously thought to be the primary source of NADPH in microalgae [33]. The expression level of glucose-6-phosphate dehydratase (*G6PDH*), which was the limiting enzyme in the PPP [34], showed a 1.73-fold increase in the DH-overexpressed strain. The results indicated that overexpression of the DH domain could elevate the NADPH supply, thereby promoting fatty acid biosynthesis.

In general, the accumulated lipids, including SFA and PUFA, are mainly in the form of triacylglycerols (TAG) [35]. TAG are primary lipid storage in microalgae and occur mainly via the TAG pathway [36]. Therefore, the increase in TAG content also represents an increase in fatty acid content. Phosphatidic acid phosphatase (PAP), lysophosphatidic acid acyltransferase (LPAAT), glycerol-3-phosphate acyltransferase (GPAT), and diacylglycerol acyltransferase (DGAT) are the key enzymes that catalyze the acylation of glycerol-3-phosphate (G3P) and lead to the production of TAGs [37–40]. Comparing the wild-type strain with the ER-overexpression strain, no significant difference in gene expression or metabolites related to TAG biosynthesis was observed. Nevertheless, the expression levels of critical genes involved in TAG biosynthesis, such as *GPAT*, *LPAAT* and *PAP,* were significantly upregulated in the DH-overexpression strain compared to the wild-type strain (Fig. 5). Increased expression of *GPAT*, *LPAAT* and *DGAT* resulted in a higher PUFA yield in *Phaeodactylum tricornutum* [41]. Additionally, the cellular metabolites involved in TAG biosynthesis (3-phosphoglyceric acid and 1-monopalmitin) were upregulated. Thus, the significantly increased expression levels of *GPAT*, *LPAAT* and *PAP* resulted in an increase in the TL and PUFA contents in the DH-overexpression strain.

### Effects of ROS changes on fatty acids biosynthesis in engineered strains

ROS can cause damage to DNA, proteins, lipids, and other biological molecules, leading to the loss of protein function and even cell death [42]. Lipids, mostly PUFAs, can be oxidized by ROS formed in aerobic environments. Conversely, lipid peroxidation can also lead to the accumulation of high levels of ROS [43]. By determining ROS levels, the time profile levels of ROS during fermentation appeared first decrease and then increase gradually. However, different changes in the intracellular ROS were found among the three strains during the fermentation process. Compared to the wild-type strain, the ROS level remained low in the DH-overexpression strain, which indicated that antioxidant (perhaps PUFAs) contents increased to induce the reduction of oxygen free radicals in the DH-overexpression strain. The expression level of genes encoding superoxide dismutase (SOD) was also upregulated in the DH-overexpression strain. SOD is one of the key enzymes in fatty acid oxidation and forms the first defense line against ROS by reducing it to H_2_O_2_ [44]. Moreover, the transcription levels of genes encoding fatty acid oxidation (such as *gala* and *aslA*) were significantly downregulated in the DH-overexpression strain. Combined with the lower concentration of ROS, these results indicated that the DH domain enhanced PUFA accumulation, probably by reducing fatty acid oxidation.

While the opposite changes were found in the ER-overexpression strain, a higher ROS level was exhibited in this strain than the wild-type strain. With the consumption of nutrition and prolonged stress, lipid peroxidation, particularly PUFA peroxidization, could result in a significant accumulation of ROS [45]. Combined with the upregulated genes involved in fatty acid oxidation (*galA* and *aslA*), these results might indicate that overexpression of the ER domain enhances lipid peroxidation, thereby reducing the content of TL and PUFA.

### Effects of other changes on fatty acid biosynthesis in engineered strains

Surprisingly, the contents of metabolites involved in lipid biosynthesis (such as palmitic acid, myristic acid and stearic acid) increased significantly in the two engineered strains, especially he ER-overexpression strain. Lipid profile analysis revealed a positive correlation between the accumulation of EPA and SFA. According to researchers, SFA and PUFA were synthesized via the conventional desaturase/elongase pathway (FAS) and PKS pathways, respectively [6]. Transcriptomic analysis revealed that the genes involved in the FAS pathway, such as 3-oxoacyl-[acyl-carrier-protein] synthase (*fabF*), elongation of very-long-chain fatty acids protein 6 (*ELO6)* and elongation of very-long-chain fatty acids protein 9 (*ELO9*) were upregulated by 1.41, 1.36 and 2.15-fold in the ER-overexpression strain, respectively. In the DH-overexpression strain, however, no such differences were observed. Our previous research demonstrated that after three days of fermentation, the proportion of DHA in total lipids remained constant, whereas the EPA content increased with the increased fermentation time [46]. Due to the absence of certain desaturases, Song et al. hypothesized that a third pathway involving both the FAS and PKS pathways in PUFA biosynthesis in *Thraustochytrids* [25]. These results might suggest that EPA accumulation is related to SFA biosynthesis, but further research is necessary [17].

## Conclusion

In the present study, to investigate the role of ORFC of the PKS gene cluster in fatty acid accumulation, ORFC was expressed heterologously in yeast. The significantly increased content of TL and PUFA indicated that ORFC was required for fatty acid biosynthesis. Subsequently, the DH and ER domains located on ORFC were overexpressed in *Schizochytrium limacinum* SR21, respectively. Lipid profile showed a significant increase in the PUFA content in the DH-overexpression strain. In the ER-overexpression strain, on the other hand, the PUFA content decreased while the SFA content increased. Additionally, metabolomic and transcriptomic analysis revealed that overexpression of the DH domain increased PPP and TAG biosynthesis while decreasing TCA and fatty acid oxidation, thereby enhancing the biosynthesis of fatty acids. However, overexpression of the ER domain appeared to enhance the TCA cycle and fatty acid oxidation during the lipid accumulation phase. As a result, the DH and ER domains of ORFC might have distinct roles in lipid accumulation. The DH domain was required for PUFA (mainly DHA) synthesis, whereas the ER domain inhibited PUFA synthesis, which might be related to the biosynthesis of SFA in *Schizochytrium limacinum* SR21. This work provides an experimental basis for clarifying the role of ORFC in lipid accumulation and theoretical support for engineering strains with high PUFA yields.

## Methods

### Strains and plasmids

The primers, plasmids and strains used in this study are listed in Additional file 1: Table S1 and Table 2. All fragments obtained by polymerase chain reaction (PCR) or overlap extension PCR were gel purified using a kit (Takara; Japan) before cloning. Fragment assembly was performed using the Gibson method [47].

**Table 2:** Strains and plasmids used in this study

Strains and plasmids	Relevant genotype	Source
*E.coli* strains		
DH5α	F-, φ80dlacZΔM15, Δ(lacZYA-argF) U169, deoR, recA1, endA1, hsdR17(rK-, mK +), phoA, supE44, λ-, thi-1, gyrA96, relA1	Takara
*S. cerevisiae* strains		
YSG50	MATα, ade2-1, ade3Δ22, ura3-1, his3-11,15, trp1-1, leu2-3,112 and can1-100	A gift from Prof. Yuan
YSG50-C	MATα, ade2-1, ade3Δ22, ura3-1, his3-11,15, trp1-1, leu2-3,112 and can1-101, orfC^+^	This study
*Schizochytrium limacinum* strains		
SR21		ATCC
DH-overexpression strain	ble^+^, DH^+^	This study
ER-overexpression strain	ble^+^, ER^+^	This study
Plasmids		
pRS426	2μ, URA3, Amp^R^	New England Biolabs
pRS426-orfC	2μ, URA3, Amp^R^, ADH1p, CYC1t, orfC	This study
pBlueZeo-MAT	Amp^R^, Zeo^R^, MAT^+^	Constructed by our lab [48]
pBlueZeo-DH	Amp^R^, Zeo^R^, DH^+^	This study
pBlueZeo-ER	Amp^R^, Zeo^R^, ER^+^	This study

Amp^R^ indicates ampicillin resistance, Zeo^R^ indicates zeocin resistance

### Media and culture conditions

The fermentation and seed broth of *Schizochytrium limacinum* SR21 was the same as that used in our previous study [48]. All media were autoclaved at 121 °C for 20 min before use. The seed medium was inoculated at 2% (v/v) and cultured in a shaker at 200 rpm and 28 °C. The cells were grown in 100 mL flasks with 20 mL of seed medium and cultivated for 48 h. After two generations of cultivation, the seed culture (4% v/v) was then transferred to 500 mL flasks with 100 mL of fermentation medium. Three parallel samples were performed.

### Overexpression of the DH/ER domain in ORFC in *Schizochytrium limacinum SR21*

Homologous recombination was used to generate overexpression strains. The DH and ER domains were amplified from the cDNA of *Schizochytrium limacinum* SR21 and ligated to the promoter TEF1p by overlap extension PCR. After digestion with BamHI and SpeI, the resulting DNA fragments were ligated into pBlueZeo-MAT which was linearized by BamHI and SpeI. Zeocin was used as the selection marker. The resulting plasmids and primers used are listed in Additional file 1: Fig. S1 and Table S1.

The overexpression plasmids were linearized by ApaI and NotI, and then transformed into *Schizochytrium limacinum* SR21 through electro-transformation according to our previous study [48]. Specifically, 100 μL of competent cells and ~ 1 μg of linearized plasmids were added to a 0.2 cm gap cuvette (Bio-Rad, California, USA) for electroporation. After electroporation, the cells were recovered at 28 °C in 200 rpm in a recovery medium (seed medium with 1 M sorbitol), and then 100 μL of cells were plated on a solid selection medium containing 2% agar and 50 μg/mL zeocin. After 3–5 days of incubation, the resulting transformants were transformed into the seed medium containing 50 μg/mL zeocin at 28 °C and 200 rpm. Genomic DNA and RNA were extracted and used for PCR and qPCR analysis, respectively.

### Quantitative real-time PCR (qPCR) analysis

Total RNA was isolated from 1 mL of *Schizochytrium limacinum* SR21 cells in exponential growth phase using a ZR Fungal/ Bacterial RNA MicroPrep kit (ZYMO, California, USA). cDNA was synthesized by HiScript III RT Supermix for qPCR (+ gDNA wiper) (Vazyme, Nanjing, China) according to the manufacturer’s protocol. qPCR was performed using ChamQ Universal SYBR qPCR Master mix (Vazyme, Nanjing, China). QTower 3G (Analytik Jena, Germany) was used to detect the expression of target genes. The ACT (actin) gene was used as a reference gene in the calculations.

### Determination of intracellular ROS

The ROS level in cells was measured using the commercialized probe 2',7'-dichlorodihydrofluorescein diacetate (DCFH-DA, Beyotime Biotechnology, China) according to the optimized manufacturer's instructions. Briefly, an aliquot of the culture was collected, centrifuged and resuspended to 1–2 × 10^6^ cells/mL in 20 mM PBS (pH 7.0). The cell suspension was mixed with diluted probes of DCFH-DA at the final concentration of 10 mM, incubated at 37 °C in the dark for 30 min, and then washed three times with 10 mM PBS. The fluorescence intensity was measured at an excitation wavelength of 488 nm and an emission wavelength of 525 nm using SpectraMax M5 (Molecular Devices, San Jose, USA).

### Determination of biomass, TL and fatty acid composition

One milliliter of fermentation broth was collected every day during the entire fermentation period by centrifugation at 8000 *g* for 5 min. The cell pellet was washed with 0.7% saline solution and dried using a vacuum freezer dryer to obtain the total biomass.

TL content and fatty acid composition were determined from 5 mL of culture according to our previous report [46]. Briefly, 5 mL of fermentation broth was mixed with 5 mL of HCl (12 mol/L) and incubated at 65 °C for 30 min. After transesterification, the mixture was extracted 5 times with 3 mL of n-hexane and evaporated to dryness by nitrogen flow. The samples were then redissolved in 5 mL of 0.5 M KOH–CH_3_OH and 5 mL of 30% BF_3_-ether and applied to a gas chromatograph (Agilent GC 7890, USA) equipped with a 100 m × 0.25 mm capillary column (SP-2560, USA). Deuterated myristic acid (Sigma, Burlington, USA) was used as an internal standard.

### GC–MS performance and metabolomics data analysis

Sample preparation was performed as described previously [46]. In brief, samples on the 3rd day of fermentation were quenched with 80% cold methanol (− 40 °C, v/v) and then centrifuged at 8000 *g* and 4 °C for 10 min. Samples were ground under liquid nitrogen, extracted with prechilled methanol (− 40 °C) and freeze-dried. The resulting extracts were used for further analysis. The methyl ester of heptadecanoic acid was used as an internal standard for quantification.

GC–MS analysis was carried out with a variation on the two-stage technique as described previously [48]. Briefly, samples were derivatized in 50 μL of 20 mg/mL methoxyamine hydrochloride in pyridine for 2 h at 37 °C. Then 60 μL of N-methyl-N-(trimethylsilyl) trifluoroacetamide (MSTFA, Sigma, Burlington, USA) was added followed by incubation for another 2 h at 37 °C. Finally, the samples were centrifuged at the maximum speed for 10 min at 4 °C. The resulting sample was analyzed on an Agilent 7890-5975C GC–MS solution system (Agilent, Sacramento, USA). First, the GC oven temperature was maintained at 85 °C for 5 min, increased to 270 °C at a rate of 15 °C/min, and held for 5 min. Electron impulse ionization was applied at 70 eV. Helium was used as the carrier gas, and the flow rate was maintained at 1 mL min^−1^. The working range of the mass spectrometer was m/z 50−600.

GC–MS data were processed based on Li’s method [46]. Each peak was determined by alignment with the mass spectra in the NIST 2.2 library (National Institute of Standards and Technology, USA). The data of the identified metabolites were normalized and analyzed using SIMCA 14.1 (Umetrics, Umeå, Sweden) for multivariate analysis. PCA and OPLS-DA were used to identify the differential metabolites of the wild-type and overexpression strains. Differential metabolites were identified with VIP value greater than 1, and *p* < 0.05 was considered as statistically significant. A heat map was generated by the pheatmap package in R [49]. Six replicates were used in this experiment. MetaMapp analysis was performed in the MetaMapp and Cytoscape software packages [50].

### RNA extraction, sequencing, and data analysis

Samples were collected at 3rd day and immediately centrifuged for 5 min at 8000 *g* and 4 ℃. The resulting pellets were frozen in liquid nitrogen and then stored at − 80 ℃ until use. Three biological replicates were prepared. Following the manufacturer's protocol, total RNA was extracted from liquid-ground cells using a TRIzol reagent kit (Invitrogen, Carlsbad, CA, USA). An Agilent 2100 Bioanalyzer (Agilent Technologies, Palo Alto, CA, USA) was used to determine RNA quality. Oligo(dT) beads were used to enrich poly(A) mRNA from total RNA. cDNA was prepared by DNA polymerase I, RNase H, dNTPs and buffer. After purification, cDNA fragments were end-repaired, poly(A) was added followed by ligation to Illumina sequencing adapters. Finally, the cDNA libraries were sequenced with Illumina HiSeq2500 by Gene Denovo Biotechnology Co. (Guangzhou, China).

Clean reads obtained from RNA-seq were aligned to the reference genome using HISAT2.2.4. Fragments per kilobase of transcript per million reads mapped (FPKM) was used to calculate the abundance and variations of assembled transcripts using Stringtie V1.3.1 [51, 52]. EdgeR was used to analyze DEGs with the criteria of |log_2_ (fold change)| > 1 in expression level and FDR value below 0.05 [53]. GO terms and KEGG pathway enrichment analysis of the DEGs were performed using the online OmicShare tools (https://www.omicshare.com/tools/). All expressed genes were used as the background.

### Heterologous expression of ORFC in *Saccharomyces cerevisiae*

To prepare the ORFC heterologous plasmid, yeast promoter ADH1p (397 bp) and terminator CYC1t (248 bp) were amplified from *S. cerevisiae* YSG50 genomic DNA. The target gene ORFC was cloned from the cDNA of *Schizochytrium limacinum* SR21. These individual fragments were assembled by overlap extension PCR [54]. After purification, the target gene was ligated with BamHI linearized pRS426 (5726 bp) (Additional file 1: Fig. S1). Then, the successfully constructed plasmid was electro-transformed in *S. cerevisiae* according to a previous method [54]. The recombinant strain was transferred to fermentation medium for 3 days at 30 ℃ and 200 rpm, and the fermentation broth was used for further fatty acid analysis (Additional file 6).

### Data analysis

Different parameters, such as total biomass and lipid profile, were statistically analyzed using R (https://www.r-project.org/) with several publicly available packages. Figures were generated with several R packages, such as pheatmap, circlize, and EasyStat. Analysis of variance (ANOVA) was used to determine the significant differences between different strains. Tukey’s multiple comparisons test was used for post hoc analysis to compare individual means. Spearman correlation analysis was used to explore the correlation among the fatty acids. Each experiment was conducted in triplicates. Values are expressed as means ± standard deviation (SD) and *p* < 0.05 was used to determine statistical significance.

## Supplementary Information

**Additional file 1: Table S1.** Primers used in this experiment.** Fig. S1.** Plasmids constructed in this study. **A** DH overexpression plasmid. **B** ER overexpression plasmid. **C** ORFC heterologous plasmid.** Fig. S2.** ORFC heterologous expression in *S. cerevisia* YSG50. **A** The ORFC heterologous expression strain was selected by the Ura-depletion plate. **B** Genomic PCR analysis of ORFC. WT indicated the wild-type strain; YSG50-C indicated the ORFC heterologous strain.** Fig. S3.** **A** Genomic PCR products of DH and ER domains in the wild-type strain. **B** Plasmids construction validation by NotI and ApaI digestion. p-ER: ER-overexpression plasmid; p-DH: DH-overexpression plasmid. **C** Genomic PCR of Zeo expression cassette. P indicates positive control, N indicates the wild-type strain, DH^+^ indicates DH-overexpression strain, ER^+^ indicates ER-overexpression strain. **Fig. S4** Gene copies of **A** DH and **B** ER domain in the wild-type and engineered strain, respectively, by qPCR analysis. All data are expressed as mean ± SD of three independent experiments.** Fig. S5.** Permutation test for the OPLS-DA model: **A** wild-type strain and DH-overexpressed strain and **B** wild-type strain and ER-overexpressed strain.** Fig. S6.** Metabolomics profiling by GC-MS reveals divergent metabolic phenotypes. **A** DH-overexpression strain compared with the wild-type strain. **B** ER-overexpression strain compared with the wild-type strain. Each node denotes an identified metabolite (red, up-regulated; blue, down-regulated;* p* < 0.05 by a two-tailed Student’s t-test). Node size reflects median fold change.** Fig. S7.** Heatmap of significant genes (log2 (fold change)) and their enriched pathways compared to the wild-type strain. Left frame: DH-overexpression strain; right frame: ER-overexpression strain. **Table S2.** Fatty acids composition analysis of domains located in ORFC overexpression strains

**Additional file 2: Table S3.** DEGs between WT vs DH^+^ and DH^+^ vs ER^+^. WT, DH^+^ and ER^+^ indicate the wild-type strain, DH-overexpression strain and ER-overexpressed strain, respectively.

**Additional file 3: Table S4.** DEGs among WT vs DH^+^, WT vs ER^+^ and DH^+^ vs ER^+^. WT, DH^+^ and ER^+^ indicate the wild-type strain, DH-overexpression strain and ER-overexpressed strain, respectively.

**Additional file 4: Table S5.** DEGs between WT vs DH^+^ and WT vs ER^+^. WT, DH^+^ and ER^+^ indicate the wild-type strain, DH-overexpression strain and ER-overexpressed strain, respectively.

**Additional file 5: Table S6. **The specific values of significant genes (log2 (fold change)) and their enriched pathways compared to the wild-type strain. WT, DH^+^ and ER^+^ indicate the wild-type strain, DH-overexpression strain and ER-overexpressed strain, respectively. FC means fold change.

**Additional file 6: Table S7. **Gene expression of DEGs in WT vs DH^+^, WT vs ER^+^ and DH^+^ vs ER^+^, respectively. WT, DH^+^ and ER^+^ indicate the wild-type strain, DH-overexpression strain and ER-overexpressed strain, respectively.

## Data Availability

All data generated or analyzed during this study are included in the article and its Additional files.

## Abbreviations

ORF: Open reading frame
PKS: Polyketide synthases
PUFA: Polyunsaturated fatty acids
SFA: Saturated fatty acids
TL: Total lipids
DHA: Docosahexaenoic acid
DPA: Docosapentaenoic acid
EPA: Eicosapentaenoic acid
ARA: Arachidonic acid
DGLA: Dihomo-gamma linolenic acid
DH: Dehydratase
ER: Enoyl reductase
ROS: Reactive oxygen species
PCA: Principal component analysis
OPLS-DA: Orthogonal partial least-squares discriminant analysis
VIP: Variable importance in the projection
PPP pathway: Pentose phosphate pathway
MVA: Mevalonate
FAMEs: Fatty acid methyl esters
FDR: False discovery rate
TAG: Triacylglycerols
GPAT: Glycerol-3-phosphate acyltransferase
LPAAT: Lysophosphatidic acid acyltransferase
PAP: Phosphatidic acid phosphatase
DGAT: Diacylglycerol acyltransferase
G3P: Glycerol-3-phosphate
SOD: Superoxide dismutase
PDH: Pyruvate dehydrogenase
CS: Citrate synthase
sucA: 2-Oxoglutarate dehydrogenase E1 component
sucB: 2-Oxoglutarate dehydrogenase E2 component
G6PDH: Glucose-6-phosphate dehydratase
fabF: 3-Oxoacyl-[acyl-carrier-protein] synthase
ELO6: Elongation of very-long-chain fatty acids protein 6
ELO9: Elongation of very-long-chain fatty acids protein 9
