# Supplementary material for: Function of ORFC of the polyketide synthase gene cluster on fatty acid accumulation in Schizochytrium limacinum SR21
Source: Biotechnol Biofuels. 2021 Jul 23;14:163. doi: 10.1186/s13068-021-02014-9 (PMC8305795; doi:10.1186/s13068-021-02014-9)
Supplement: Supplementary file 1 — Additional file 1: Table S1. Primers used in this experiment. Fig. S1. Plasmids constructed in this study. A DH overexpression plasmid. B ER overexpression plasmid. C ORFC heterologous plasmid. Fig. S2. ORFC heterologous expression in S. cerevisia YSG50. A The ORFC heterologous expression strain was selected by the Ura-depletion plate. B Genomic PCR analysis of ORFC. WT indicated the wild-type strain; YSG50-C indicated the ORFC heterologous strain. Fig. S3. A Genomic PCR products of DH and ER domains in the wild-type strain. B Plasmids construction validation by NotI and ApaI digestion. p-ER: ER-overexpression plasmid; p-DH: DH-overexpression plasmid. C Genomic PCR of Zeo expression cassette. P indicates positive control, N indicates the wild-type strain, DH+ indicates DH-overexpression strain, ER+ indicates ER-overexpression strain. Fig. S4 Gene copies of A DH and B ER domain in the wild-type and engineered strain, respectively, by qPCR analysis. All data are expressed as mean ± SD of three independent experiments. Fig. S5. Permutation test for the OPLS-DA model: A wild-type strain and DH-overexpressed strain and B wild-type strain and ER-overexpressed strain. Fig. S6. Metabolomics profiling by GC-MS reveals divergent metabolic phenotypes. A DH-overexpression strain compared with the wild-type strain. B ER-overexpression strain compared with the wild-type strain. Each node denotes an identified metabolite (red, up-regulated; blue, down-regulated; p < 0.05 by a two-tailed Student’s t-test). Node size reflects median fold change. Fig. S7. Heatmap of significant genes (log2 (fold change)) and their enriched pathways compared to the wild-type strain. Left frame: DH-overexpression strain; right frame: ER-overexpression strain. Table S2. Fatty acids composition analysis of domains located in ORFC overexpression strains [file 13068_2021_2014_MOESM1_ESM.docx]

**Function of** **ORFC of the polyketide synthase gene cluster on fatty acid accumulation in *Schizochytrium limacinum* SR21**

Yanyan Shi^1^, Zhen Chen^2^, Yixin Li^1^, Xingyu Cao^1^, Lijie Yang^1^, Yiyuan Xu^1^, Zhipeng Li^3^, Ning He^1, *^

^1^ Department of Chemical and Biochemical Engineering, College of Chemistry and Chemical Engineering, Xiamen University, Xiamen 361005, China

^2^ College of Life Science, Xinyang Normal University, Xinyang 464000, China

^3^ College of Food and Biological Engineering, Jimei University, Xiamen 361005, China

* Corresponding author

College of Chemistry and Chemical Engineering, Xiamen University, Xiamen 361005, China.

E-mail address: hening@xmu.edu.cn.

**Table S1**. Primers used in this experiment.

| Gene Name | Sequence (5'-3') | Product length |
| --- | --- | --- |
| DH-ApaI-1 | gggccctctcaaagattaaggca | 2431bp |
| DH-2 | GCAGAGTTTGTACATagttcctcaccttgtcgtattatactatgccgatata |  |
| DH-3 | caaggtgaggaactATGTACAAACTCTGCGCCCG | 439bp |
| DH-SpeI-4 | gactagtTTAGAAAGTCTGGCCCTTCT |  |
| ER-BamHI-1 | taatttgcctgcagcccggggcgggatccgatccccc | 518bp |
| ER-2 | CCTTCATCACCCATagttcctcaccttgtcgtattatactatgccgata |  |
| ER-3 | aaggtgaggaactATGGGTGATGAAGGCTTCATGAAG | 949bp |
| ER-SpeI-4 | gactagtTTAGCCCTGGACGGGAACAT |  |
| Zeo-F | AAACCATGGCCAAGTTGACC | 375bp |
| Zeo-R | CCTGCTCCTCGGCCACGAAG |  |
| qDH-F | CCCGTTGTGATCCACCGCAAG | 103bp |
| qDH-R | GCAGCCCACAGCAATGGTCA |  |
| qER-F | TCGCCGTCAACCTCATCCACT | 93bp |
| qER-R | CCTCGACGACAGTAACGCCCTT |  |
| ADH1p-F | agcttgatatcgaattcctgcagcccggggCGGATATCCTTTTGTTGTTTCCG | 397bp |
| ADH1p-R | gctcctccttggtcatctcccagAGTTGATTGTATGCTTGGTATAGC |  |
| ORFC-F | gctataccaagcatacaatcaactCTGGGAGATGACCAAGGAGGAGC | 3908bp |
| ORFC-R | gctcctccttggtcatctcccagAGGAGGCATGGAGTCGAAGGAGT |  |
| CYC1t-F | actccttcgactccatgcctcctCTGGGAGATGACCAAGGAGGAGC | 248bp |
| CYC1t-F | cggtggcggccgctctagaactagtggatcGCAAATTAAAGCCTTCGAGC |  |
| qORFC-F | GCTCTCTGTTGATGGTGTAGTC | 87bp |
| qORFC-R | AGACCAGTCTGGGAGATGAA |  |

Note: sequences which underlined were restriction enzyme sites. The lowercase letters indicated overlap nucleic acid base.





**Fig. S1** Plasmids constructed in this study. (A) DH overexpression plasmid. (B) ER overexpression plasmid. (C) ORFC heterologous plasmid.





**Fig. S2** ORFC heterologous expression in *S. cerevisia* YSG50. (A) The ORFC heterologous expression strain selected by the Ura-depletion plate. (B) Genomic PCR analysis of ORFC. WT indicated the wild-type strain; YSG50-C indicated the ORFC heterologous strain.





**Fig. S3** (A) Genomic PCR products of DH and ER domains in the wild-type strain. (B) Plasmids’ construction validation by NotI and ApaI digestion. p-ER: ER-overexpression plasmid; p-DH: DH-overexpression plasmid. (C) Genomic PCR of Zeo expression cassette. P indicates positive control, N indicates the wild-type strain, DH^+^ indicates DH-overexpression strain, ER^+^ indicates ER-overexpression strain.





**Fig. S4** Relative expression of DH and ER domain in the wild-type and engineered strain by qPCR analysis. All data are expressed as mean ± SD of three independent experiments.





**Fig. S5** Permutation test for the OPLS-DA model: (A) wild-type strain and DH-overexpressed strain and (B) wild-type strain and ER-overexpressed strain.


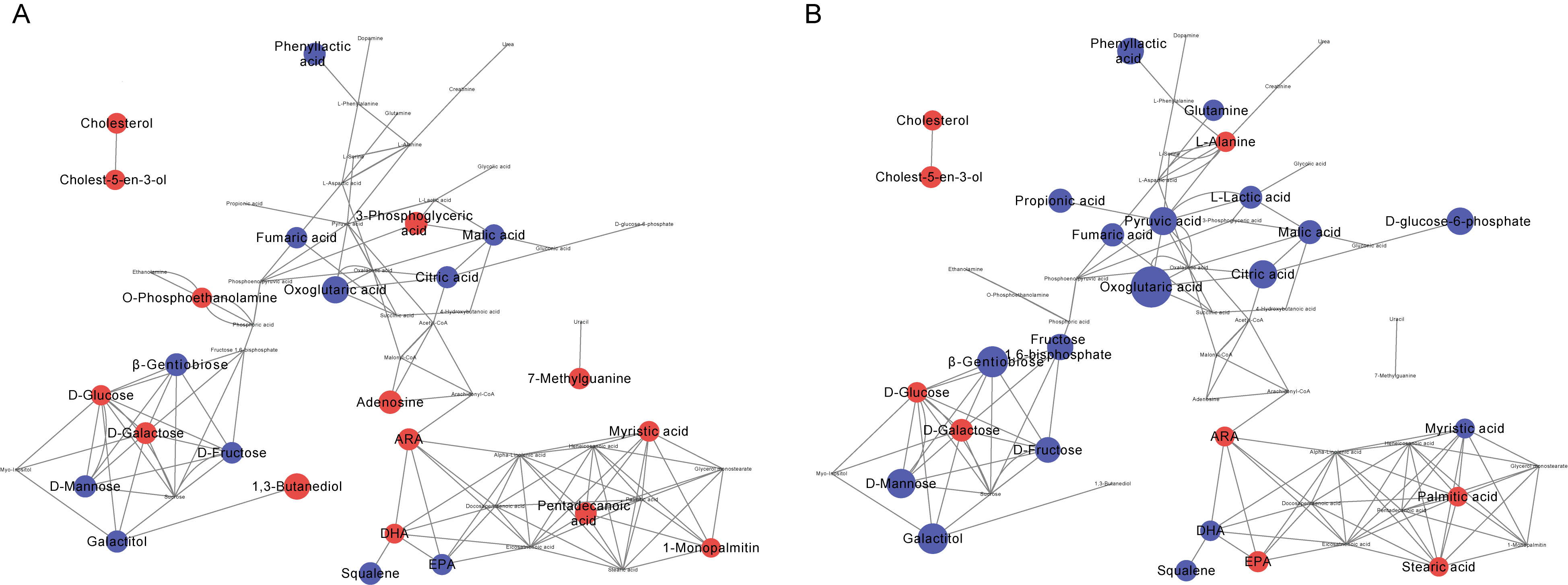


**Fig. S6** Metabolomics profiling by GC-MS reveals divergent metabolic phenotypes. (A) DH-overexpression strain compared with the wild strain. (B) ER-overexpression strain compared with the wild strain. Each node denotes an identified metabolite (red, up-regulated; blue, down-regulated; *p* < 0.05 by a two-tailed Student’s t-test). Node size reflects median fold change.





**Fig. S7** Heatmap of significant genes (log_2_ (fold change)) and their enriched pathways compared to the wild-type strain. Left frame: DH-overexpression strain; right frame: ER-overexpression strain.

**Table S2** Fatty acids composition analysis of domains located in ORFC overexpression strains

| Lipid profile (%) | WT | DH1^+^ | DH2^+^ | ER^+^ |
| --- | --- | --- | --- | --- |
| TL/Biomass(g/g) | 0.58±0.01 | 0.65±0.02* | 0.59±0.04 | 0.51±0.01* |
| C12:0 | 0.13±0.00 | 0.12±0.01 | 0.13±0.00 | 0.11±0.01 |
| C14:0 | 4.73±0.15 | 4.53±0.10 | 4.37±0.15 | 3.91±0.19 |
| C16:0 | 50.18±0.31 | 49.01±0.62 | 51.33±0.22 | 56.29±0.53* |
| C18:0 | 1.87±0.12 | 1.86±0.08 | 1.70±0.02 | 1.14±0.06* |
| C20:0 | 0.33±0.02 | 0.26±0.01 | 0.32±0.02 | 0.09±0.00* |
| DGLA | 0.43±0.02 | 0.50±0.04 | 0.44±0.04 | 0.26±0.01* |
| EPA | 0.48±0.03 | 0.37±0.01* | 0.52±0.03 | 0.76±0.04* |
| DPA | 6.52±0.35 | 6.90±0.21 | 6.02±0.03 | 5.40±0.26* |
| DHA | 30.62±1.54 | 32.62±0.59* | 29.03±0.44 | 24.18±0.88* |
| PUFA/SFA | 0.67±0.05 | 0.73±0.02* | 0.62±0.01 | 0.50±0.04* |

Note: * indicates *p* < 0.05. WT, DH1^+^, DH2^+^ and ER^+^ represent the wild-type strain, the first DH domain overexpression strain, the second DH domain overexpression strain, and the ER-overexpression strain, respectively. TL: total lipids; DGLA: dihomo-gamma linolenic acid; EPA: eicosapentaenoic acid; DPA: docosapentaenoic acid; DHA: docosahexaenoic acid; PUFA: polyunsaturated fatty acids; SFA: saturated fatty acids.
